# The Effect of On-Site Potentials on Supratransmission in One-Dimensional Hamiltonian Lattices

**DOI:** 10.3390/e25030423

**Published:** 2023-02-26

**Authors:** Tassos Bountis, Jorge E. Macías-Díaz

**Affiliations:** 1 Department of Mathematics, University of Patras, 26500 Patras, Greece; 2 Center for Integrable Systems, P. G. Demidov Yaroslavl State University, 150003 Yaroslavl, Russia; 3 Department of Mathematics and Didactics of Mathematics, School of Digital Technologies, Tallinn University, Narva Rd. 25, 10120 Tallinn, Estonia; 4 Departamento de Matemáticas y Física, Universidad Autónoma de Aguascalientes, Avenida Universidad 940, Ciudad Universitaria, Aguascalientes 20100, Mexico

**Keywords:** system with long-range interactions, on-site potential, supratransmission, Hamiltonian lattices, 65M06, 65M12

## Abstract

We investigated a class of one-dimensional (1D) Hamiltonian *N*-particle lattices whose binary interactions are quadratic and/or quartic in the potential. We also included on-site potential terms, frequently considered in connection with localization phenomena, in this class. Applying a sinusoidal perturbation at one end of the lattice and an absorbing boundary on the other, we studied the phenomenon of supratransmission and its dependence on two ranges of interactions, 0<α<∞ and 0<β<∞, as the effect of the on-site potential terms of the Hamiltonian varied. In previous works, we studied the critical amplitude $A_{s}\left(\alpha ,\Omega \right)$ at which supratransmission occurs, for one range parameter α, and showed that there was a sharp threshold above which energy was transmitted in the form of large-amplitude nonlinear modes, as long as the driving frequency Ω lay in the forbidden band-gap of the system. In the absence of on-site potentials, it is known that $A_{s}\left(\alpha ,\Omega \right)$ increases monotonically the longer the range of interactions is (i.e., as α⟶0). However, when on-site potential terms are taken into account, $A_{s}\left(\alpha ,\Omega \right)$ reaches a maximum at a low value of α that depends on Ω, below which supratransmission thresholds *decrease* sharply to lower values. In this work, we studied this phenomenon further, as the contribution of the on-site potential terms varied, and we explored in detail their effect on the supratransmission thresholds.

## 1. Introduction

Supratransmission was first investigated in chains of coupled nonlinear oscillators [1], when the first oscillator is forced sinusoidally at a frequency in the so-called forbidden band gap, so that the excitation of linear normal modes is avoided. The theoretical prediction of the critical driving amplitude, above which the boundary begins to transmit energy, was first established experimentally, and later approximated analytically, using known solutions of the sine-Gordon equation [2]. Further studies showed that this phenomenon is also present in well-known nonlinear models, such as the classical Fermi–Pasta–Ulam–Tsingou (FPUT) chain [3] and the Klein–Gordon and double sine-Gordon models [4]. Various investigations have led to the development of reliable computational techniques to approximate the occurrence of this phenomenon [5,6]. In this direction, various works have been devoted to the design and analysis of novel numerical methods using variational properties, while new computational methodologies have been proposed based on these approaches [7].

Initially, most studies focused on mathematical models with only nearest neighbor interactions. However, more recently, new results have emerged focusing on global interactions with a long range of applicability [8,9,10]. Indeed, globally interacting systems have led to a better understanding of nonlinear problems in physics [11]. More generally, it is known that systems with long-range interactions (LRIs) can be expressed as Riesz-type fractional models in the continuous limit [12,13,14]. Such approaches provide a convenient bridge between spatially discrete and continuous systems, and they lead to a better understanding of globally interacting systems [15,16].

Regarding the study of supratransmission in globally interacting systems, its presence has been established in Riesz space fractional sine-Gordon equations [17], fractional models of Josephson transmission lines [18] and in FPUT chains with different ranges of interactions [19]. More recently, a general 1D Hamiltonian lattice consisting of quadratic global interactions, which included an on-site potential (of the Klein–Gordon type), was investigated in [20], where it was found that supratransmission was also present in that system. However, the critical amplitude $A_{s}\left(\alpha \right)$ in that model exhibited a surprising nonmonotonic behavior: as expected, it occurred at *higher* amplitudes the *longer* the range of interactions, but reached a maximum at a value $\alpha =\alpha_{max}$ that depended on Ω!. Below this value, supratransmission thresholds *decreased* sharply to values lower than the nearest neighbor α=∞ limit.

In [21], the authors considered a different Hamiltonian form of the model studied in [20], in which on-site potentials were combined with LRIs of purely quartic order. One of the reasons was to examine whether the presence of quadratic interactions and their associated linear spectrum are needed for supratransmission, as suggested by the seminal work in [1]. The conclusion in [21] was that this feature does *not* constitute a necessary condition for supratransmission. However, as in the case of [20], the critical amplitudes $A_{s}\left(\alpha \right)$ again exhibited the same behavior of reaching a maximum at α≈2 that depended on the value of Ω, and then as α⟶0 fell to values even below their α=∞ limit.

In this work, we investigated the dependence of critical supratransmission amplitudes on two LRI scale parameters, α and β (of the quadratic and quartic interactions, respectively), as well as the frequency of oscillations Ω. To this end, we employed a 1D Hamiltonian lattice with both quadratic and quartic interactions, in which the on-site potential terms were gradually switched off, as a multiplicative parameter c>0 tended to zero. The results were quite surprising and revealed the role of the driving frequency Ω on the critical supratransmission amplitudes of these lattices as the on-site potential terms were gradually switched off.

The paper is organized as follows. In Section 2, we introduce our Hamiltonian model and its associated equations of motion in the form of an initial–boundary value problem. Our Hamiltonian system, in its general form, describes globally interacting particles with both quadratic and quartic interactions, as well as on-site terms of the sine-Gordon type. Section 4 describes the fully discretized model we used to approximate the solutions of the equations of motion and the Hamiltonian functional. In Section 5, we describe the computational experiments we performed to study the occurrence of supratransmission in three cases: Case I with both quartic and quadratic LRIs, Case II with only quartic LRIs and Case III with only quadratic LRIs, in the limit where the on-site potentials vanish. Next, in Section 6 we study the dependence of supratransmission amplitudes on different values of α and β, determining ranges of interactions. Finally, in Section 7 we end our study with a discussion of our results and concluding remarks.

## 2. Mathematical Model

We consider *N*-particle one-dimensional Hamiltonian lattices, with N∈N sufficiently large, whose N+2 particles are governed by the Hamiltonian

(1) $$\begin{array}{cc} H & =\sum_{n=1}^{N}\left(\frac{1}{2}p_{n}^{2}+cV\left(x_{n}\right)\right)+\frac{a^{2}}{2M_{1}}\sum_{n=0}^{N}\sum_{m=n+1}^{N+1}\frac{{\left(x_{m}-x_{n}\right)}^{2}}{{\left(m-n\right)}^{\alpha }}\\ \; & \;+\frac{b^{2}}{4M_{2}}\sum_{n=0}^{N}\sum_{m=n+1}^{N+1}\frac{{\left(x_{m}-x_{n}\right)}^{4}}{{\left(m-n\right)}^{\beta }} \end{array}$$

where α and β are non-negative real numbers (including *∞*) determining the range of pair particle interactions, V:R→R is a continuously differentiable function representing the on-site potential of the system and $a,b,c\in R^{+}\cup \left\{0\right\}$ are constants. We also use the scaling factors

(2) $$M_{1}=\frac{1}{N+1}\sum_{n=0}^{N}\sum_{m=n+1}^{N+1}\frac{1}{{\left(m-n\right)}^{\alpha }},\;\;\;M_{2}=\frac{1}{N+1}\sum_{n=0}^{N}\sum_{m=n+1}^{N+1}\frac{1}{{\left(m-n\right)}^{\beta }},$$

to ensure that all terms in our Hamiltonians are *extensive*, i.e., proportional to the number of particles *N*, see [20] and references therein. The constants a≥0, b≥0 are fixed as follows: in Case I, both a,b are nonzero; in Case II a=0 and b≠0; and in Case III a≠0b=0. In all cases, the parameter c≥0 is taken to gradually tend to zero.

Each $x_{n}\left(t\right):R\to R$ is a function such that $x_{n}\left(t\right)\in C^{2}\left(R\right)$, for each n∈{1,…,N}, and $p_{n}\left(t\right)$ is the time derivative of $x_{n}\left(t\right)$. In this context, we define, respectively, the kinetic and potential energy of the *n*th particle by

(3) $$K_{n}=\frac{1}{2}p_{n}^{2}$$

and

(4) $$\begin{array}{cc} P_{n} & =cV\left(x_{n}\right)+\frac{a^{2}}{2M_{1}}\sum_{m=n+1}^{N+1}\frac{{\left(x_{m}-x_{n}\right)}^{2}}{{\left(m-n\right)}^{\alpha }}+\frac{b^{2}}{4M_{2}}\sum_{m=n+1}^{N+1}\frac{{\left(x_{m}-x_{n}\right)}^{4}}{{\left(m-n\right)}^{\beta }}. \end{array}$$

The on-site potential function $V(x_{n})$ is taken to be of the sine-Gordon type, V(u)=1−cos(u). Alternatively, we can also use the Klein–Gordon potential

(5) $$V\left(u\right)=\frac{1}{2!}u^{2}-\frac{1}{4!}u^{4}+\frac{1}{6!}u^{6}.$$

Clearly, the values of α=β=∞ represent the case of nearest neighbor interactions. If α,β∈[0,1], the mutual particle interactions have comparable contributions and the system is said to have *long-range interactions* (LRIs). The case where α,β∈(1,∞), is said to correspond to *short-range interactions* (SRIs).

Thus, the equations of motion associated with our Hamiltonian (1) are as follows:

(6) $$\begin{array}{c} \begin{array}{cc} {\ddot{x}}_{n} & =\frac{a^{2}}{M_{1}}\sum_{\begin{array}{c} m=1\\ m\neq n \end{array}}^{N}\frac{x_{m}-x_{n}}{{\left|m-n\right|}^{\alpha }}+\frac{b^{2}}{M_{2}}\sum_{\begin{array}{c} m=1\\ m\neq n \end{array}}^{N}\frac{{\left(x_{m}-x_{n}\right)}^{3}}{{\left|m-n\right|}^{\beta }}-cV^{\prime }\left(x_{n}\right)-\gamma_{n}{\dot{x}}_{n}, \end{array}\\ \mathrm{with}\;b.c.\;\left\{\begin{array}{cc} x_{0}\left(t\right)=A\sin\left(\Omega t\right), & \forall t\geq 0,\\ x_{N+1}\left(t\right)=x_{N}\left(t\right), & \forall t\geq 0,\\ x_{n}\left(0\right)={\dot{x}}_{n}\left(0\right)=0, & \forall n\in \{1,\ldots ,N+1\}, \end{array}\right. \end{array}$$

where a “damping” term is added at each node (with coefficient $\gamma_{n}>0$), simulating an absorbing boundary at the right end of the system. This avoids the generation of returning shock waves at the right end of the chain [22,23]. In fact, it is convenient to use

(7) $$\gamma_{n}=0.5\left[1+\tanh\left(\frac{2n-N_{0}+N}{6}\right)\right],$$

for each n∈{1,…,N+1}, assuming that $N_{0}<N$.

A simpler form of this model was investigated in [20], which corresponds to (6) with b=0. An interesting question, therefore, arises as to whether supratransmission is still present if the interaction terms in the potentials are quartic instead of quadratic (i.e., a=0). As was shown in [21], supratransmission neither requires the presence of harmonic interactions, nor the absence of on-site terms in the potential, but it is rather a generic phenomenon present in this class of systems.

Regarding the role of the damping term in (6), we note that for relatively large *N*, we let $1\ll N_{0}\ll N$. Thus, the form of (7) guarantees that the nodes before $N-N_{0}$ will have a damping coefficient that is approximately equal to zero. On the other hand, the $\gamma_{n}$ monotonically increase near the right end of the chain, thus ensuring that the waves reflected back in the system are rapidly attenuated, see [24]. We have found empirically that if $N_{0}\approx N/4$ wave reflection at the right boundary is effectively negligible; thus, in all our simulations we used N=100 and $N_{0}=25$.

## 3. Calculation of the Phonon Spectrum

It is important to determine the phonon (or forbidden) band of frequencies for our system, so as to be able to interpret the results of our computations in later sections. To this end, we considered the linear terms in the position variables on the right-hand side (RHS) of (6) and obtained an NxN symmetric matrix, whose eigenvalues were difficult to obtain analytically for general values of α and *N*.

Note, however, that in the limit α→0 and large *N*, we have $M_{1}=N/2$, while our symmetric matrix has a multiplicative factor $2a^{2}/N$, with diagonal terms equal to $-N(1+c/2a^{2})+1$ and all off-diagonal terms equal to 1. Fortunately, this matrix can be diagonalized and has the eigenvalues −c and $-(c+2a^{2})$ (multiplicity N−1). Now, for α>0 (and small), all the terms that were unity in the matrix slightly decrease, which suggests that the phonon frequencies become non-degenerate and lie within an interval approximated by [$\sqrt{c}$, $\sqrt{c+2a^{2}}$].

## 4. Numerical Methodology

The solutions of system (6) will be approximated using an explicit finite difference scheme, which possesses similar Hamiltonian properties as our mathematical model and efficiently approximates the solutions of (6) over the time interval [0,T], for large *T*.

We first fix a uniform partition

(8) $$0=t_{0}<t_{1}<\ldots <t_{j}<\ldots <t_{K}=T$$

of the interval [0,T], with τ sufficiently small. Next, we define the time step $t_{j+\frac{1}{2}}=t_{j}+\frac{1}{2}\tau$, for each j∈{0,1,…,K−1}, set $x_{n}^{j}=x_{n}\left(t_{j}\right)$, for n∈{0,1,…,N+1} and j∈{0,1,…,K}, and introduce the discrete linear operators

(9) $$\begin{array}{cc} \mu_{t}x_{n}^{j} & =\frac{1}{2}\left(x_{n}^{j+1}+x_{n}^{j}\right), \end{array}$$

(10) $$\begin{array}{cc} \delta_{t}^{\left(1\right)}x_{n}^{j} & =\frac{x_{n}^{j+1}-x_{n}^{j}}{\tau }, \end{array}$$

(11) $$\begin{array}{cc} \delta_{t}^{\left(2\right)}x_{n}^{j} & =\frac{x_{n}^{j+1}-2x_{n}^{j}+x_{n}^{j-1}}{\tau^{2}}, \end{array}$$

and

(12) $$\begin{array}{cc} \delta_{t,x}V\left(x_{n}^{j}\right) & =\left\{\begin{array}{cc} \frac{V\left(x_{n}^{j+1}\right)-V\left(x_{n}^{j}\right)}{x_{n}^{j+1}-x_{n}^{j}}, & \mathrm{if}\;x_{n}^{j+1}\neq x_{n}^{j},\\ V^{\prime }\left(x_{n}^{j+\frac{1}{2}}\right), & \mathrm{if}\;x_{n}^{j+1}=x_{n}^{j}. \end{array}\right. \end{array}$$

Thus, the fully discretized form of (6) is given by the following system of equations:

$$\mu_{t}\delta_{t}^{\left(2\right)}x_{n}^{j}=\frac{a^{2}}{M_{1}}\sum_{\begin{array}{c} m=1\\ m\neq n \end{array}}^{N}\frac{\mu_{t}\left(x_{m}^{j}-x_{n}^{j}\right)}{{\left|m-n\right|}^{\alpha }}+\frac{b^{2}}{M_{2}}\sum_{\begin{array}{c} m=1\\ m\neq n \end{array}}^{N}\frac{\mu_{t}{\left(x_{m}^{j}-x_{n}^{j}\right)}^{3}}{{\left|m-n\right|}^{\beta }}-c\delta_{t,x}V\left(x_{n}^{j}\right)-\gamma_{n}\delta_{t}^{\left(1\right)}x_{n}^{j},$$

(13) $$\begin{array}{c} \mathrm{such}\;\mathrm{that}\;\left\{\begin{array}{cc} x_{0}^{j}=A\sin\left(\Omega t_{j}\right), & \mathrm{if}\;0\leq j\leq K,\\ x_{N+1}^{j}=x_{N}^{j}, & \mathrm{if}\;0\leq j\leq K,\\ x_{n}^{0}=x_{n}^{1}=x_{n}^{2}=0, & \mathrm{if}\;1\leq n\leq N. \end{array}\right. \end{array}$$

It follows that the discrete form of the Hamiltonian functional is given by the following:

(14) $$\begin{array}{cc} H^{j} & =\sum_{n=1}^{N}\left[\frac{1}{2}\left(\delta_{t}^{\left(1\right)}x_{n}^{j}\right)\left(\delta_{t}^{\left(1\right)}x_{n}^{j-1}\right)+cV\left(x_{n}^{j}\right)\right]+\frac{a^{2}}{2M_{1}}\sum_{n=0}^{N}\sum_{n=n+1}^{N+1}\frac{{\left(x_{m}^{j}-x_{n}^{j}\right)}^{2}}{{\left(x-n\right)}^{\alpha }}\\ \; & \;+\frac{b^{2}}{4M_{2}}\sum_{n=0}^{N}\sum_{n=n+1}^{N+1}\frac{{\left(x_{m}^{j}-x_{n}^{j}\right)}^{4}}{{\left(x-n\right)}^{\beta }}, \end{array}$$

for each j∈{1,…,K−1}. Thus, the discrete kinetic and potential energies of the *n*th particle at time $t_{j}$ are given, respectively, by

(15) $$K_{n}^{j}=\frac{1}{2}\left(\delta_{t}^{\left(1\right)}x_{n}^{j}\right)\left(\delta_{t}^{\left(1\right)}x_{n}^{j-1}\right),$$

and

(16) $$\begin{array}{cc} P_{n} & =cV\left(x_{n}^{j}\right)+\frac{a^{2}}{2M_{1}}\sum_{n=n+1}^{N+1}\frac{{\left(x_{m}^{j}-x_{n}^{j}\right)}^{2}}{{\left(x-n\right)}^{\alpha }}+\frac{b^{2}}{4M_{2}}\sum_{n=n+1}^{N+1}\frac{{\left(x_{m}^{j}-x_{n}^{j}\right)}^{4}}{{\left(x-n\right)}^{\beta }}. \end{array}$$

This method is an adaptation of a four-step scheme that approximates the solutions of Riesz space fractional hyperbolic partial differential equations with Hamiltonian structures [25]. It uses spatial step sizes equal to 1 and is both stable and quadratically convergent [23].

In the following sections, we describe how we conducted a series of computational experiments to study the behavior of the critical amplitude $A_{s}$ for supratransmission in 1D lattices governed by (6), for various values of a,b and range parameters α and β. For comparison purposes, we employed the approach followed in [20] for 1D lattices consisting of N=100 particles. Regarding the absorbing boundary, we let $N_{0}=25$ and set τ=0.05 in all our simulations. Moreover, we used the sine-Gordon on-site potential, i.e.,

(17) V(x)=1−cosx,∀x∈R.

## 5. Computational Results

The purpose of this section is to carry out simulations in order to study the behavior of critical amplitudes for supratransmission as a function of the model parameters, particularly c≥0 measuring the contribution of the on-site potential terms. Throughout, we consider N=100 nodes and final time T=100. The computation time step everywhere is τ=0.05.

### 5.1. Case I for Different Frequencies

**Figure 1:**
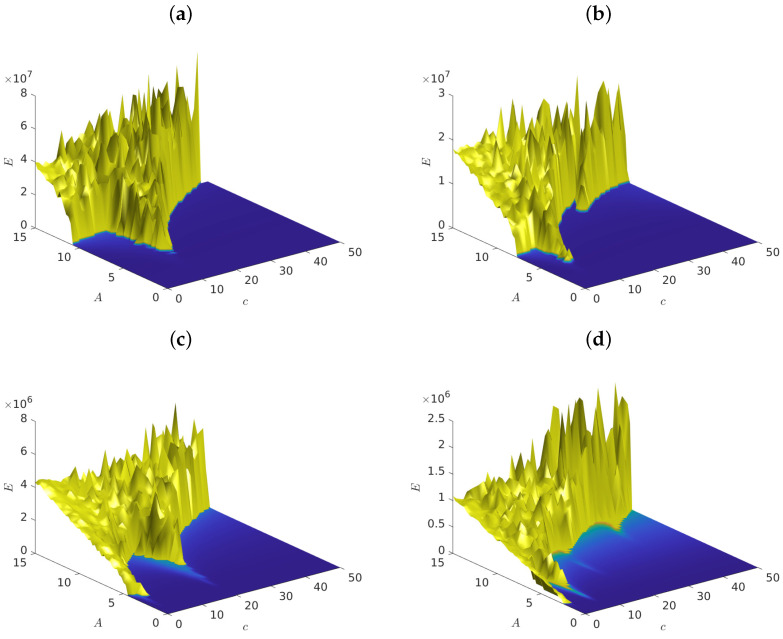
Graphs of the total energy of system (6) versus the driving amplitude *A* and *c*, for (**a**) Ω=3.5, (**b**) Ω=2.5, (**c**) Ω=1.5 and (**d**) Ω=0.9. We used α=β=0, fixed a=b=1 and the time step size was equal to 0.05.

As our first example, we considered the Case I parameters a=b=1, with maximal LRIs α=β=0, and selected various values for the driving frequency Ω. Under these circumstances, Figure 1 shows the graph of the total energy (6) (measured at the steady state of the system) as a function of the driving amplitude *A* and the coefficient *c*, for the following values of the frequencies: (a) Ω=3.5, (b) Ω=2.5, (c) Ω=1.5 and (d) Ω=0.9.

Let us first observe in this figure the presence of supratransmission in all cases, as exemplified by the sudden, upward surge of the energy from the blue E=0 level of inactivity to the yellow/green “mountain ranges” at higher values of *A*. Note now in Figure 1a that, as *c* decreased, the critical amplitude $A_{s}\left(c\right)$ for supratransmission experienced a minimum around c=10 and then increased to a higher value as *c* tended to zero. A similar picture also occurs in Figure 1b with the minimum of $A_{s}$ located near c=5.

According to the analysis in Section 3, the phonon band in this case was $\left[\sqrt{c},\sqrt{c+2}\right]$, which yielded [3.16,3.46] for (a) and [2.23,2.65] for (b), which are close to the driving frequencies Ω=3.5 and Ω=2.5 respectively. A similar observation regarding the first minimum also held for Figure 1c,d, where the phonon bands were [1.4,2 and [1,1.73], respectively. Regarding the secondary minima in the last two figures, we suggest that they correspond to higher order resonances. For example, the second minimum in Figure 1c occurred at c≈19, which would excite a $\sqrt{19}\approx 4.5=3\Omega$ resonance.

### 5.2. Case II for Different Frequencies

We next examined a Hamiltonian (1) where the quadratic interactions were absent, setting a=0 and b=1 and allowing the range parameters to vary from 0<α=β up to α=β=9 and higher. Remarkably, the results obtained, for the same frequency values as considered in Case I, led to figures that *differed very little* from Figure 1 (and hence are not plotted here) provided we kept the same frequency values used in the plots of Figure 1.

**Figure 2:**
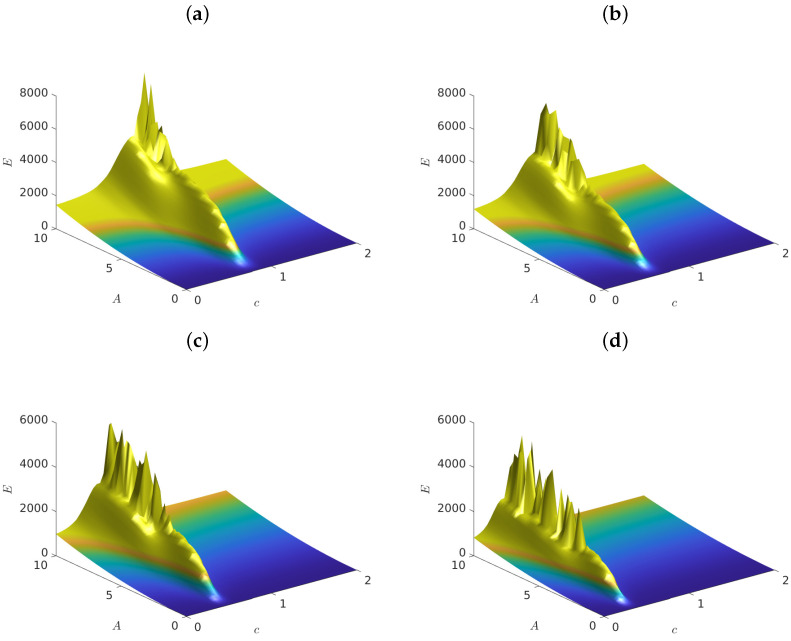
Graphs of the total energy of system (6) versus $A_{s}$ and *c*, for (**a**) Ω=0.9, (**b**) Ω=0.8, (**c**) Ω=0.7 and (**d**) Ω=0.6. We used α=β=0, and fixed a=0 and b=1.

Next, we proceeded to study, in Figure 2, the same Case II example with a=0 and b=1, with all other parameters the same as in Figure 1. The important difference here was that we chose driving frequencies *smaller than 1* in the different plots: (a) Ω=0.9, (b) Ω=0.8, (c) Ω=0.7 and (d) Ω=0.6.

The difference between the results of Figure 1 and Figure 2 was striking: the energy landscapes for these frequencies were dramatically smoother, and the $A_{s}$ values exhibited a single minimum, $0<c_{min}<1$, whose value diminished monotonically as Ω decreased. Interestingly, since the only quadratic term in the potential was $cx_{n}^{2}$, the minimum of the $A_{s}$ was smaller than 1 and decreased as Ω decreased. Below this minimum, $A_{s}$*significantly increased* as c→0, which meant that the lattice became more resilient against supratransmission in the absence of on-site potential terms.

### 5.3. Case III: Absence of Quartic Terms

**Figure 3:**
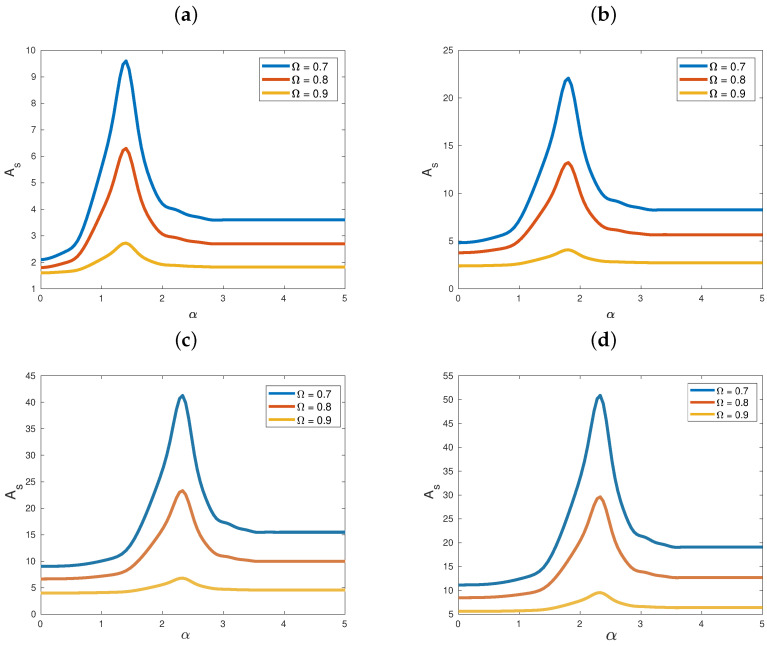
Graphs of the supratransmission threshold $A_{s}$ as a function of α, for various values of Ω (see legends). We let a=2, b=0, N=100, T=200 and V(x)=1−cosx. Various values of *C* were employed, namely (**a**) c=1, (**b**) c=0.8, (**c**) c=0.4 and (**d**) c=0.2.

Next, we turn to an example of Case III, with a=2 and b=0, and examine in Figure 3 the behavior of critical amplitudes $A_{s}$ as functions of the range parameter α, for diminishing c=1,0.8,0.4,0.2. In all plots of this figure we used frequencies Ω<1, and observed no supratransmission whatsoever. One might think, therefore, that this was due to resonances with the phonon band and would not occur if we used Ω>1. However, when we did apply higher driving frequencies, all we observed was harmonic waves traveling down the lattice, showing no sudden increase in their amplitudes. This suggested that quartic interactions in the potential were crucial for the occurrence of supratransmission.

**Figure 4:**
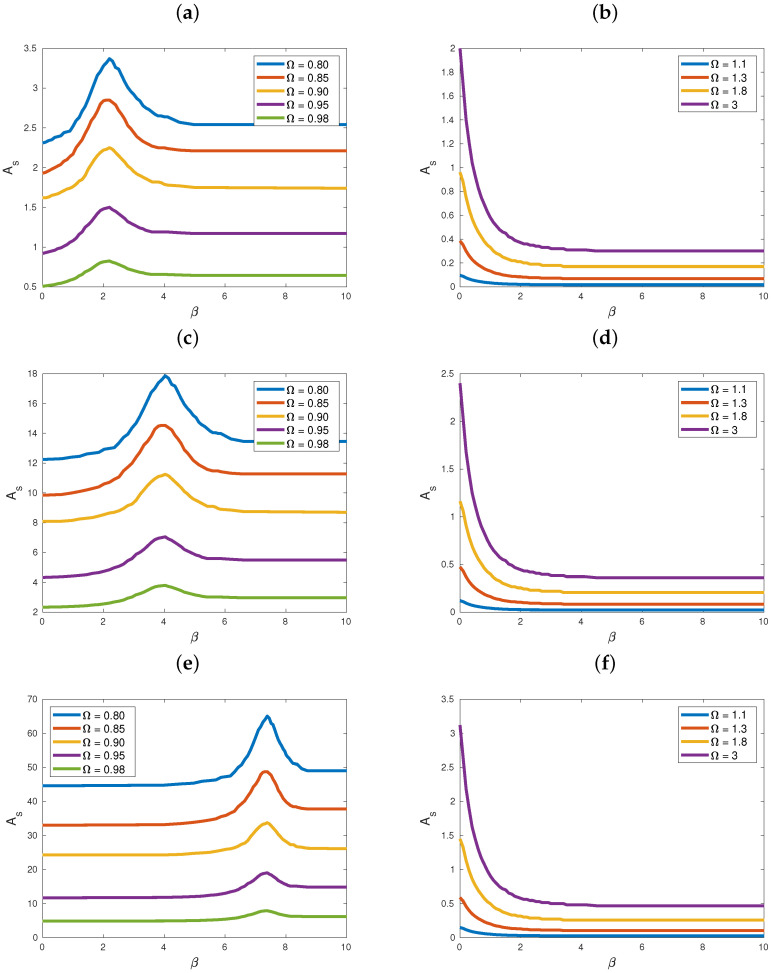
Graphs of the supratransmission threshold $A_{s}$ as a function of the parameter β, using various values of Ω. We let a=0, b=4, N=100, T=200 and V(x)=1−cosx. Decreasing values of *c* were employed, namely c=0.9 (**top row**), c=0.4 (**middle row**) and c=0.2 (**bottom row**). The subfigures in (**a**–**f**) give the value of Ω that corresponds to the curve of the figure with the same color. In the right column figures, all critical amplitudes $A_{s}$ attained finite values at β=0, which increased linearly with Ω.

In the next section, where we discuss a case in which quartic interaction terms were present, the plots of Figure 3 are similar to those of Figure 4a,c,e, for Ω<1, and supratransmission was absent. However, when Ω>1 in that system, as Figure 4b,d,f show, resonances with the phonon band were avoided and supratransmission did occur!

## 6. A Case II Study with Different β Parameters

Finally, we examined the dependence of critical amplitudes $A_{s}$ on the range parameter β, for different values of the driving frequency Ω. We did this for Case II of our study, corresponding to a=0, b=4 and examined the behavior of the results as the value of *c* is decreased, plotting the results of our computations in Figure 4.

Observe first the general behavior of $A_{s}$ vs. β for Ω<1 in Figure 4a,c,e: note the presence of a local maximum in the graphs, which shifted to higher values of β as *c* diminished from 0.9 (top row) to 0.4 (middle row) and 0.2 (bottom row). Clearly, for these frequencies, the location of the maximum increased for higher β. Note first that, in all these figures, all critical amplitudes *increased* as *c* decreased, in agreement with the results of previous sections.

By contrast, in Figure 4b,d,f, the behavior of $A_{s}$ vs. β monotonically increased as β tended to zero, converging to constant values, which increased as *c* diminished. This was very similar to what we already encountered in Figure 3b,d and was in agreement with the results of Section 3, as there were no resonances with the phonon band in this case; hence, supratransmission occurred in this case also, with the $A_{s}$ values rising linearly with Ω as β→0.

## 7. Discussion and Conclusions

The process of supratransmission has become very popular in recent years since its discovery nearly two decades ago. The main reason for this is due to its appeal as a mechanism that allows one to transmit a large amount of energy through a nonlinear medium by simply driving one end of the medium by Asin(Ωt) at the appropriate frequencies and amplitudes. Thus, it has inspired many authors due to its obvious applications in many physical situations.

Almost all studies so far have concentrated on one-dimensional (1D) chains of nonlinear oscillators, as a first step towards understanding supratransmission in higher dimensions. An additional advantage of 1D systems is the fact that, at least under nearest neighbor coupling, they have been extensively analyzed for more than 60 years, numerically as well as analytically. Thus, the first papers on supratrasmission focused on a number of classical conservative 1D models, for which a continuum limit analysis yielded partial differential equations that connected supratransmission to nonlinear wave solutions of the systems and, thus, permitted the derivation of analytical formulas connecting $A_{s}$ and Ω to the emergence of such solutions.

More recently, a number of papers appeared verifying that supratransmission is also ubiquitous in models that involve long-range interactions (LRIs) among the particles by multiplying the interaction between the *n*th and *m*th particles by a factor of the form $1/{\left(n-m\right)}^{\alpha }$, with 0≤α≤∞. In this context, 0≤α≤1 represents LRIs, while short range interactions (SRIs) correspond to the α>1 values, with α=∞ being the nearest neighbor case. The authors of the present paper and their collaborators analyzed in detail the occurrence of supratransmission in 1D systems using the FPUT 1D lattice as an example [9,19], and they later included in their studies the effect of on-site terms in the potential [20].

In the absence of on-site potentials, one finds that $A_{s}\left(\alpha ,\Omega \right)$ grows monotonically the longer the range of interactions (i.e., as α⟶0). However, when on-site potential terms are introduced, $A_{s}\left(\alpha ,\Omega \right)$ is seen to attain a maximum at low α values depending on Ω, below which supratransmission thresholds sharply decrease. In this paper, we made a first attempt to further explore these phenomena by introducing a parameter c>0 before the on-site potential, and letting c⟶0.

We first examined the case where both quadratic and quartic global LRIs were present and found that, the higher Ω>1 is, the clearer it became that as c⟶0, supratransmition critical amplitudes increased significantly. This picture became even clearer when only quartic terms were included in the potential. Next, we examined the dependence of critical amplitudes on the range parameters, α and β, in a Case II and a Case III study for one set of frequencies with Ω<1 and one with Ω>1, for decreasing values of *c*. When Ω<1, in both studies, $A_{s}$ had a single maximum at a β value that increased as *c* decreased, hence no supratransmission. However, for Ω>1, in the case where quartic interaction terms were present, supratransmission did occur, as there was no local maximum, and the $A_{s}$ monotonically increased at β=0 values, which also grew steadily as c⟶0.

These findings were explained by carrying out an analysis of the phonon band of our system. This allowed us to explain the reason why supratransmission failed to occur, as the range parameters α and β decreased, due to resonances of the driving frequency with the phonons, while it did occur when Ω lay above the phonon band.

It would, of course, be desirable to connect analytically the occurrence of supratransmission with the excitation of specific nonlinear solutions of the problem, as has been conducted in a number of references, where it was possible to derive a PDE in the continuum limit, see, e.g., [3,4]. This was mainly accomplished in the continuum limit of 1D lattices with nearest neighbor interactions, which appeared difficult to extend to the LRI systems studied in this paper. Perhaps it could be conducted for cases where *a fractional PDE* can be derived for our system, but this is a matter that we would prefer to address in a future publication.

We also looked at snapshots of the energy distribution along the chain to guess the type of nonlinear excitation activated at the supratransmission state. Interestingly, the graphs we obtained strongly resembled a *travelling breather*, similar to what one finds in the continuum limit of a lattice with only nearest neighbor interactions! It would thus be very interesting, in a future publication, to attempt to find an analogous analytical solution and study its stability for a lattice that involves long range interactions, such as with the one studied in the present paper.

In closing, we believe that the results we have described in this paper provide a motivation to search for physical arguments to justify and perhaps explain our findings. One main result is that the presence of on-site potentials often hinders the occurrence of supratransmission. The relationships we have uncovered between the fundamental parameters of our system and the phenomenon of supertransmission are interesting, and, in our opinion, quite remarkable. They, therefore, merit further study before we can say that we understand this phenomenon and we are ready to investigate it in higher dimensional settings.

## Data Availability

Not applicable.
